# Development of HIV-Resistant CAR T Cells by CRISPR/Cas-Mediated CAR Integration into the *CCR5* Locus

**DOI:** 10.3390/v15010202

**Published:** 2023-01-10

**Authors:** Frederik Holm Rothemejer, Nanna Pi Lauritsen, Anna Karina Juhl, Mariane Høgsbjerg Schleimann, Saskia König, Ole Schmeltz Søgaard, Rasmus O. Bak, Martin Tolstrup

**Affiliations:** 1 Department of Clinical Medicine, Aarhus University, 8200 Aarhus, Denmark; 2 Department of Infectious Diseases, Aarhus University Hospital, 8200 Aarhus, Denmark; 3 Department of Biomedicine, Aarhus University, 8200 Aarhus, Denmark; 4 Aarhus Institute of Advanced Studies, Aarhus University, 8200 Aarhus, Denmark

**Keywords:** HIV, CAR T cells, CRISPR/Cas9, humanized mice

## Abstract

Adoptive immunotherapy using chimeric antigen receptor (CAR) T cells has been highly successful in treating B cell malignancies and holds great potential as a curative strategy for HIV infection. Recent advances in the use of anti-HIV broadly neutralizing antibodies (bNAbs) have provided vital information for optimal antigen targeting of CAR T cells. However, CD4+ CAR T cells are susceptible to HIV infection, limiting their therapeutic potential. In the current study, we engineered HIV-resistant CAR T cells using CRISPR/Cas9-mediated integration of a CAR cassette into the *CCR5* locus. We used a single chain variable fragment (scFv) of the clinically potent bNAb 10-1074 as the antigen-targeting domain in our anti-HIV CAR T cells. Our anti-HIV CAR T cells showed specific lysis of HIV-infected cells in vitro. In a PBMC humanized mouse model of HIV infection, the anti-HIV CAR T cells expanded and transiently limited HIV infection. In conclusion, this study provides proof-of-concept for developing HIV-resistant CAR T cells using CRISPR/Cas9 targeted integration.

## 1. Introduction

Adoptive immunotherapy using chimeric antigen receptor (CAR) T cells has been successfully used against refractory B cell malignancies [1,2,3]. Immunological control of tumors and chronic infections share many challenges, making CAR T cells an attractive approach for the eradication of HIV-infected cells [4]. Although several anti-HIV CAR T cells have entered clinical trials, the field still faces many obstacles, including efficient antigen targeting and susceptibility to infection of the infused CAR T cell products [5,6,7,8,9,10].

Recent advances in recombinant antibody cloning techniques have led to the discovery of several potent anti-HIV-1 broadly neutralizing antibodies (bNAbs) that are being explored in HIV prevention and cure-related clinical trials [11]. These bNAbs bind to highly conserved epitopes on the viral envelope (Env) protein. In clinical trials, several recombinant bNAbs have been able to prolong the time to viral rebound after stopping ART [12,13,14,15]. bNAbs in the form of single chain variable fragments (scFv) have been used as the antigen-targeting domain in CAR T cell constructs where they can specifically redirect CAR T cells to target HIV-infected cells with Env cell surface expression [16,17,18].

Several studies have highlighted the importance of combining CD4+ and CD8+ T cells to enhance the potency of CAR T cell products [19,20,21,22]. However, as the main target of HIV, infused CD4+ CAR T cells attracted to an HIV-infected cell risk becoming infected, thus limiting their therapeutic benefit [8,23]. Gene editing tools have allowed for selective disruption of CCR5, a co-receptor necessary for R5-tropic HIV to enter cells, thereby rendering the cells resistant to HIV infection [24,25,26,27,28]. This approach has been explored in vivo using either zing finger-nucleases or megaTAL nucleases to induce CCR5 disruption [24,25,27], NTC03617198. The CRISPR/Cas9 system is a more simple and cost-effective alternative to these gene editing tools and allows for highly specific targeted integration of a transgene into a specific locus by homology-directed repair (HDR) [29]. Targeted integration of a transgene by CRISPR/Cas9 can thus cause disruption of the targeted gene while integrating the transgene [30].

Here, we have produced CAR T cells using an scFv from the highly potent V3-loop bNAb 10-1074. We utilized the CRISPR/Cas9 system to integrate a CAR expression cassette into the *CCR5* locus, thereby achieving simultaneous CAR integration and CCR5 disruption, rendering the HIV-specific CAR T cells resistant to HIV infection.

## 2. Materials and Methods

### 2.1. CAR Design, Vector Cloning and AAV Production

We cloned a plasmid containing an EF1α promotor, the CAR, and the tNGFR reporter, separated by a self-cleaving T2A peptide, followed by the bGHpA signal, all flanked by 400 bp homology arms of either the *AAVS1* or *CCR5* locus. The construct was cloned into a pAAV backbone containing the ITRs of AAV2. The CAR comprised a GM-CSFRa leader sequence, an scFv from either the HIV-specific bNAb 10-1074 or the anti-CD19 scFv FMC63, followed by the hinge, transmembrane, and intracellular regions of CD28 and the CD3ζ intracellular domain.

AAV6 was produced and purified as previously described [31]. Briefly, HEK293T cells were transfected via polyethylenimine transfection with the plasmid expressing the CAR gene and pDGM6, which carried the AAV6 cap genes, AAV2 rep genes, and Ad5 helper genes (pDGM6 was a gift from David Russell; Addgene plasmid #110660). After 72 h of incubation, cells were lysed by three freeze-thaw cycles, treated with turbonuclease (Sigma-Aldrich, Burlington, MA, USA, final concentration 200 U/mL), and purified by iodixanol gradient centrifugation for 2 h at 48,000 rpm. AAV was isolated from the 40–58% iodixanol interface, concentrated using a 100 kDa Amicon filter, and stored at −80 °C. The titer of AAV6 was determined by ddPCR as previously described [32], using two primer/probe pairs, one targeting the left ITR (FW primer: 5′-GGAACCCCTAGTGATGGAGTT-3′, RV primer: 5′-CGGCCTCAGTGAGCGA-3′, probe: 5′-CACTCCCTCTCTGCGCGCTCG-ZEN-IBFQ-3′) and one targeting the bGHpA (FW primer: 5′-GCCAGCCATCTGTTGT-3′, RV primer 5′-GGAGTGGCACCTTCCA-3′, probe: 5′-HEX-TCCCCCGTGCCTTCCTTGACC-ZEN-IBFQ-3′), preceded by a nuclease treatment step to remove residual plasmid DNA.

### 2.2. Cell Lines and Virus

HEK293T cells (ATCC CRL-3216) were maintained in DMEM (Biowest, Nuaillé, France) supplemented with 10% HI-FBS (Serana, Brandenburg, Germany) and 1% P/S (Biowest, Nuaillé, France) at 37 °C under 5% CO_2_. R5-tropic HIV-eGFP reporter virus was generated using a pBR-HIV1 M NL4-3 92TH14-12 plasmid kindly provided by Martin Roelsgaard Jakobsen, Department of Biomedicine, Aarhus University [33]. HxB2D was obtained through the NIBSC Program EVA Centra for AIDS Reagents from R. Gallo and M. Popovic.

### 2.3. CAR T Cell Production

PBMCs were isolated from de-identified healthy donor buffy coats by Ficoll (GE Healthcare, Chicago, IL, USA) density separation. Primary CD4+ or CD8+ T cells were isolated from PBMCs by negative magnetic selection (Miltenyi, Bergisch Gladbach, Germany). All cytotoxicity assays and in vivo experiments were performed using CD8+ CAR T cells and CD4+ CAR T cells were used when specifically stated. T cells were then activated using CD3/CD28 antibodies (StemCell, Vancouver, BC, Canada) and cultured for 72 h in X-Vivo 15 media (StemCell, Vancouver, BC, Canada) with 5 % FBS, IL-2 (Gibco, Waltham, MA, USA), and IL-15 (Peprotech, Cranbury, NJ, USA). For genome editing of T cells, RNP complexes were generated by incubating sgRNAs (containing 2′-O-methyl-3′-phosporothioate chemical modifications [34]) targeting either *AAVS1* (5′-GGGGCCACTAGGGACAGGAT-3′, Synthego, Menlo Park, CA, USA) or *CCR5* (5′-GCAGCATAGTGAGCCCAGAA-3′, Synthego, Menlo Park, CA, USA) with Cas9 protein (Alt-R S.p. Cas9 Nuclease V3, Integrated DNA Technologies, Newark, NJ, USA) at a molar ratio of 2.5:1 (sgRNA:Cas9) at 25 °C for 10 min. 1 × 10^6^ T cells were resuspended in P3 buffer (Lonza, Basel, Switzerland) with complexed RNP and electroporated using the Lonza 4D Nucleofector (program EO-115). RNP-treated cells were then transduced with AAV6 at MOI 50.000. After 24 h, the media was changed and the edited cells were reactivated and cultured for 7 days, maintaining a density of cells at 1 × 10^6^/mL.

### 2.4. INDEL Quantification and Allele Editing Frequencies

sgRNA editing efficiency was determined by INDEL analysis (ICE online tool, Synthego, Menlo Park, CA, USA). Genomic DNA from edited and unedited CD4+ and CD8+ T cells was extracted (QuickExtract, Lucigen, Middleton, WI, USA), and the *CCR5* locus was amplified by PCR (FW primer: 5′-GCACAGGGTGGAACAAGATGG-3′, RV primer: CACCACCCCAAAGGTGACCGT-3′) and Sanger sequenced using the FW primer.

Successful CAR integration into the *CCR5* locus was determined by a duplex in-out ddPCR assay. Genomic DNA from edited cells was mixed with two primer/probe sets, one reference set (FW primer: 5′-AAGCTATGCAGGTGACAG-3′, RV primer: 5′-AGGTAGTTTCTGAACTTCTCC-3′, probe: 5′-ATGCAGCAGTGCGTCATCCC-HEX-ZEN-IBFQ-3′), and one set with the forward primer upstream of the left homology arm and the reverse primer and probe located in the EF1α promotor in the CAR construct (FW primer: 5′-GCTAGCAGCAAACCTTCC-3′, RV primer: CTTCTCGGGGACTGTG-3′, probe: 5′-CCCACTGACGGGCACCGGA-FAM-ZEN-IBFQ-3′). Droplets were generated using a Bio-Rad automated droplet generator (Bio-Rad, Hercules, CA, USA), PCR amplified using a Bio-Rad thermal cycler, and analyzed using a Bio-Rad QX200 droplet reader. The integration frequencies were determined by the ratio of double positive droplets to all reference positive droplets.

### 2.5. Infection of CAR T Cells

After transduction, CAR T cells were activated with CD3/CD28 antibodies (ImmunoCult, StemCell, Vancouver BC, Canada) for 48 h. Then, cells were spinoculated with eGFP-HIV MOI 0.1 at 1250× *g* for 2 h and incubated with virus overnight. Infection was analyzed as eGFP expression by flow cytometry (MACSQuant 16, Miltenyi, Bergisch Gladbach, Germany).

### 2.6. Flow Cytometry and Antibodies

For analysis of CAR and CCR5 expression, mouse anti-human CD271-BV421 (BD Bioscience, San Jose, CA, USA, cat. No. 562562) and mouse anti-human CD195-PE (BD 560932) antibodies were used. For analysis of humanized mice, the following antibodies were used: mouse anti-human CD3-BUV395 (BD 564001), mouse anti-human CD4-BUV496 (BD 564651), mouse anti-human CD271-BV421 (BD 562562), mouse anti-human CD19-BV605 (BD 562653), mouse anti-human human-CD45-PerCP (BioLegend, San Diego, CA, USA, cat. No. 368506), rat anti-mouse murine-CD45-PE-Dazzle594 (BioLegend 103146), and mouse anti-human CD8-APC-R700 (BD 565192). Briefly, cells were incubated with TruStain FcX (BioLegend, San Diego, CA, USA) and stained for 30 min on ice. Red blood cells were lysed (FACS Lysing solution, BD, San Jose, CA, USA) and cells were washed and fixed in 1% PFA before analysis with a BD LSRFortessa X20 flow cytometer.

### 2.7. Cytotoxicity Assays

Killing of B cells was determined by isolating cryopreserved autologous B cells via negative magnetic selection (Miltenyi, Bergisch Gladbach, Germany), and then staining the B cells with CellTrace Violet (BD Bioscience, San Jose, CA, USA). CAR T cells (Effector) and B cells (Target) were co-cultured for 24 h at an effector to target (E:T) ratio of 5:1 or the specified E:T ratio. After 24 h, viability dye (NearIR Live/Dead, Invitrogen, Waltham, MA, USA) was added to the culture, incubated for 5 min, and the cells were analyzed by flow cytometry (MACSQuant 16, Miltenyi, Bergisch Gladbach, Germany).

Killing of HIV-infected CD4+ T cells was determined by isolating cryopreserved autologous CD4+ T cells through negative magnetic selection (Miltenyi, Bergisch Gladbach, Germany), followed by activation using PHA (Remel, Lenexa, KS, USA, final concentration 1 μg/mL) for 48 h. Cells were spinoculated with eGFP-HIV MIO 0.1 at 1250 × *g* for 2 h and incubated with virus overnight. CAR T cells and HIV-infected CD4+ T cells were co-cultured at an E:T ratio of 1:1 for 24 h and analyzed by flow cytometry.

Inhibition of viral replication in PBMCs was determined by depleting CD8+ T cells from cryopreserved autologous PBMCs through positive magnetic selection (Miltenyi, Bergisch Gladbach, Germany), followed by activation using PHA (Remel, Lenexa, KS, USA) for 48 h. Next, cells were spinoculated with HxB2D MOI 0.1 at 1250 × *g* for 2 h and incubated with virus overnight. CAR T cells and HIV-infected PBMCs were co-cultured in three separate co-cultures at an E:T ratio of 1:1. After 0, 72, and 120 h, supernatants from one of the three co-cultures were collected. Levels of p24 in the supernatant were determined by a standard p24 ELISA (Aalto Bioreagents, Dublin, Ireland).

### 2.8. Humanized Mouse Model of HIV Infection

All animal research was conducted according to a protocol approved by the Danish Animal Experiment Expectorate (approval 2019-15-0201-00369). NOD/SCID/IL-2Rγtrunc (NOG) female mice (8- to 10-weeks-old, Taconic, Germantown, NY, USA) were inoculated by i.p. injection of 20 × 10^6^ PBMCs, which were isolated by Ficoll (GE Healthcare, Chicago, IL, USA) density separation from healthy donor buffy coats. Fresh PBMCs were used for CAR T cell experiments. In optimization, cryopreserved PMBCs or PBMCs depleted of CD8+ T cells were used prior to infusion when indicated. CAR T cells were produced as described above from the same donor. Seven days after inoculation, CD4+ T cells were isolated from cryopreserved PBMCs from the same donor through negative magnetic selection (Miltenyi, Bergisch Gladbach, Germany), followed by PHA activation (Remel, Lenexa, KS, USA) for 48 h. CD4+ T cells were spinoculated with eGFP-HIV MOI at 1250× *g* for 2 h and incubated with virus overnight. The level of infection was determined by eGFP expression immediately prior to infusion. Nine days after inoculation of the mice, CAR T cells and 0.5 × 10^6^ HIV-infected CD4+ T cells were injected i.p. at E:T 25:1. Blood samples were taken once every week and humanization (ratio of human CD45 to murine CD45) and cell composition were analyzed by flow cytometry (Methods Section 2.6). Viral load was quantified as previously described [35]. Briefly, RNA was purified from plasma (Macherey-Nagel, Dueren, Germany) with an added nuclease step to remove cell-associated or proviral HIV DNA (Macherey-Nagel, Dueren, Germany). Then, cDNA was synthesized (Invitrogen, Waltham, MA, USA, primer: 5′-TGCCAATATTCTGTCCACCA-3′), followed by ddPCR quantification of viral load (FW primer: 5-AGGGCAGCATAGAGCAAAAA-3′, RV primer: 5′-CAAAGGAATGGGGGTTCTTT-3′, probe: 5′-6FAM-ATCCCCACTTCAACAGATGC-3′) using a Bio-Rad QX200 droplet reader.

### 2.9. Statistical Analysis

Statistical analyses were performed using GraphPad Prism software (GraphPad, San Diego, CA, USA, version 9.4.1). Flow cytometry analyses were performed using FlowJo software (FlowJo, Ashland, OR, USA, version 10.7.1). All plotted data represent means and error bars represent SEM. Comparisons of means were calculated using either two-way ANOVA, paired or unpaired t-test, and using Welch’s correction where appropriate. Nonparametric tests were used for data presented on a logarithmic axis. Fold changes were tested against the hypothesis of mean = 1. All *p* values were calculated as two-tailed and considered statistically significant when *p* < 0.05.

## 3. Results

### 3.1. Integration of a CAR into CCR5 Leads to CCR5 Disruption and Resistance to HIV Infection

**Figure 1:**
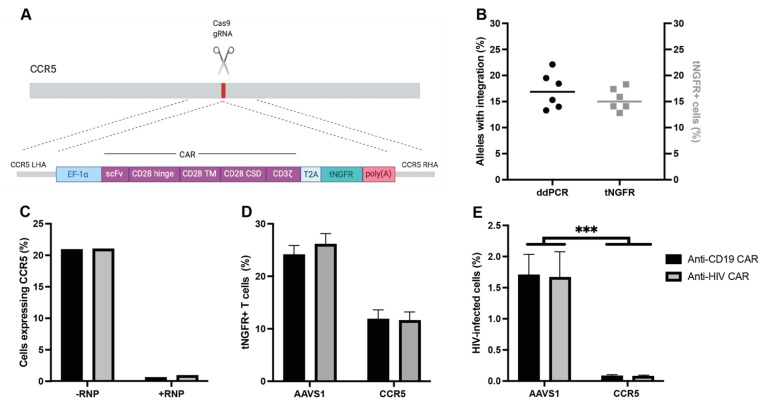
CRISPR-Cas9 integration of a CAR into *CCR5* leads to an HIV-resistant phenotype: (**A**) schematic of the CAR construct; (**B**) comparison of CAR genes specifically integrated into *CCR5* by ddPCR seven days after transduction (**left** axis) and tNGFR expression by flow cytometry four days after transduction (**right** axis); (**C**) CCR5 expression by flow cytometry in cells treated with or without CCR5-sgRNA/Cas9 RNP and transduced with CCR5-CAR AAV6; (**D**) integration rates of either anti-CD19 CAR or anti-HIV CAR into either *AAVS1* or *CCR5*; (**E**) levels of eGFP expression in CD4+ CAR T cells 24 h after infection with eGFP-HIV (data presented as means ± SEM, ***: *p* ≤ 0.001).

We generated an anti-HIV CAR construct using an scFv from bNAb 10-1074 with a CD28 co-stimulatory domain and 2A peptide-linked tNGFR as a reporter. We also generated an anti-CD19 CAR construct targeting B cells as a control, using FMC63 as the scFv, as both this target and scFv are well established [1,2,3] (Figure 1A). By using the CRISPR/Cas9 system, the CAR expression cassettes were specifically integrated into either the *CCR5* locus or the safe-harbor locus *AAVS1*.

HDR-mediated integration of the CAR into *CCR5* resulted in specific integration with comparable levels of edited alleles and tNGFR expression as measured by ddPCR and flow cytometry, respectively, for both the anti-HIV and anti-CD19 CAR (Figure 1B). CCR5-RNP-treated cells had 69% INDELs at the target site and exhibited a phenotypical disruption of CCR5 (0.79% of cells expressing CCR5 with RNP-treatment, 21% of cells expressing CCR5 without RNP-treatment) (Figure S1A and Figure 1C). The target specificity of the CAR construct (HIV Env or CD19) did not affect the integration frequency in T cells at either locus, but the integration efficiency was higher for constructs targeting the *AAVS1* than *CCR5* locus (Figure 1D). In contrast to *AAVS1*-integrated CAR T cells, *CCR5*-integrated CAR T cells had complete protection against infection following challenge with infectious HIV, irrespective of the CAR construct specificity (Figure 1E).

### 3.2. Anti-CD19 CAR T Cells Selectively Kill B Cells and Anti-HIV CAR T Cells Selectively Kill HIV-Infected Cells In Vitro

**Figure 2:**
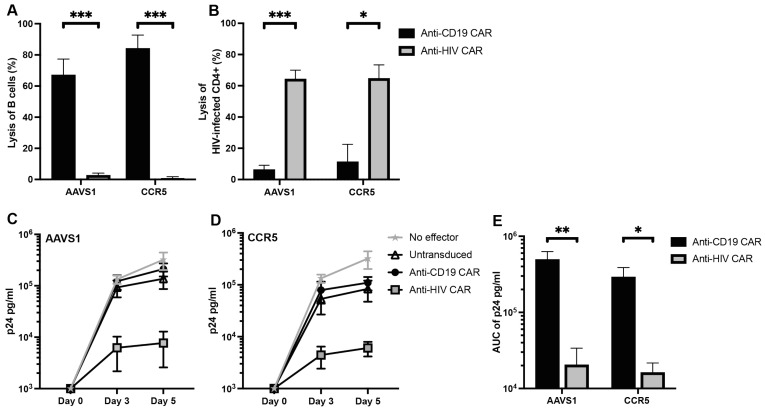
Anti-HIV CAR T cells specifically kill HIV-infected cells in vitro: (**A**) cytotoxicity assay of autologous B cells at E:T 5:1; (**B**) cytotoxicity assay of HIV-infected autologous CD4+ T cells at E:T 1:1; (**C**,**D**) levels of p24 in supernatants following five day co-culture of CAR T cells and HIV-infected CD8-depleted autologous PBMCs at E:T 1:1. (**C**): CARs are integrated into *AAVS1*, (**D**) CARs are integrated into *CCR5*; (**E**) AUC of p24 curves in (**C**,**D**) (data presented as means ± SEM, *: *p* ≤ 0.05, **: *p* ≤ 0.01, ***: *p* ≤ 0.001).

To assess the cytotoxic capacity of the CAR T cells, we first incubated CD8+ CAR T cells with autologous CD19+ B cells, one of the most frequently used targets in CAR T cell therapies. After 24 h of co-culture at an E:T ratio of 5:1, the anti-CD19 CAR T cells effectively killed the B cells with no significant difference in killing capacity between integration loci (67% and 84% for *AAVS1* and *CCR5*, respectively, *p* = 0.2331) (Figure 2A). Additionally, the anti-CD19 CAR T cells killed the B cells in a dose-dependent manner (Supplementary Figure S1B). Importantly, the anti-HIV CAR T cells did not kill the B cells demonstrating specificity of the CAR targeting domain (Figure 2A).

Next, we incubated CD8+ CAR T cells with HIV-infected autologous CD4+ T cells at an E:T ratio of 1:1. We observed specific lysis of the HIV-infected cells by the anti-HIV CAR T cells in contrast to no effect by the anti-CD19 CAR T cells (*p* = 0.0005 and *p* = 0.0202 for *AAVS1* and *CCR5*, respectively), with no difference in lysed cells between integration loci (Figure 2B).

To assess the anti-viral capacity of the CAR T cells in PBMCs, we incubated CD8+ CAR T cells with HIV-infected autologous PBMCs depleted of CD8+ T cells at an E:T ratio of 1:1. Supernatants were collected immediately after co-culture, after three, and after five days and analyzed for HIV p24 protein. After five days, the anti-HIV CAR T cell cultures had 96% (*p* = 0.0079) and 95% (*p* = 0.0286) reductions in p24 levels for *AAVS1* and *CCR5*, respectively, compared to anti-CD19 CAR T cell cultures (Figure 2C,D). Furthermore, anti-HIV CAR T cells reduced the AUC of p24 in the cultures during the five-day culture compared to anti-CD19 CARs (*p* = 0.0079 and *p* = 0.0286 for *AAVS1* and *CCR5*, respectively), with no significant difference between loci (Figure 2E).

These results demonstrated that the anti-HIV CAR T cells had no off-target activity towards B cells, effectively lysed HIV-infected cells, and limited HIV infection and spread in vitro. Furthermore, there was no difference in killing capacity between CAR T cells with the CAR expression cassette integrated into either *AAVS1* or *CCR5*, indicating no adverse effect on killing capacity by the HIV-resistant phenotype demonstrated in the CCR5-CAR T cells.

### 3.3. Anti-CD19 CAR T Cells Kill B Cells in PBMC Humanized Mice

To evaluate the CAR T cells in vivo, we established a PBMC humanized mouse model of HIV infection (modified from ref. [36,37,38]). Because this model utilized PBMCs from healthy donors, the CAR T cells could be evaluated in humanized mice derived from autologous PBMCs. We detected humanization in 83.3% of mice with up to 70% of cells in the peripheral blood being of human origin after five weeks, with no effect of using cryopreserved or CD8-depleted PBMCs prior to inoculation (Figure S2A,B). The majority of the mice (62.5%) showed signs of Graft Versus Host Disease (GVHD) 3–5 weeks after PBMC inoculation. Due to the onset of GVHD, we limited the analysis of CAR T cells to three weeks after inoculation.

**Figure 3:**
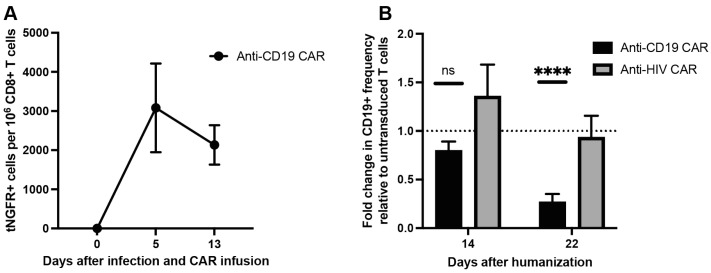
Anti-CD19 CAR T cells kill B cells in vivo: (**A**) frequency of anti-CD19 CAR T cells based on the frequency of tNGFR+ cells per 1 × 10^6^ CD8+ T cells; (**B**) fold change in the frequency of CD19+ cells for each CAR T cell-treated group normalized to the frequency of CD19+ cells in mice treated with untransduced CD8+ T cells (n = 7–8 mice from 2 biological donors in each group, data presented as means ± SEM, ns: *p* > 0.05, ****: *p* ≤ 0.0001).

We next treated humanized mice with CAR T cells by injecting donor-matched CD8+ CAR T cells or untransduced CD8+ T cells 9 days after PBMC inoculation. All CARs were *AAVS1*-integrated due to the higher integration efficiency at this locus. After five days, anti-CD19 CAR T cells were detectable in the peripheral blood (mean 3082 tNGFR+ cells per 1 × 10^6^ CD8+ T cells, 95% CI 400.9–5764) (Figure 3A). The frequency of anti-CD19 CAR T cells declined, though non-significantly, 13 days after CAR T cell infusion (mean 2134 tNGFR+ cells per 1 × 10^6^ CD8+ T cells, 95% CI 944.5–3323) (Figure 3A).

Five days after CAR T cell infusion, the frequency of B cells in the peripheral blood of either CAR T cell-treated mice did not differ significantly compared to mice treated with untransduced T cells (Figure 3B). However, 13 days after CAR T cell-infusion, the anti-CD19 CAR T cell-treated mice had a 73.1% reduction in the frequency of B cells relative to mice treated with untransduced T cells (*p* < 0.0001, 95% CI 53.5–92.7). Importantly, this decrease in B cell frequency was not observed in mice treated with anti-HIV CAR T cells (Figure 3B).

These results demonstrated that the anti-CD19 CAR T cells effectively killed B cells in vivo and that the CAR construct and CRISPR/Cas9-mediated integration of the CAR expression cassette was functionally viable for production of potent CAR T cells.

### 3.4. Anti-HIV CAR T Cells Expand and Transiently Limit HIV Infection in Humanized Mice

We infused untreated CD4+ T cells infected with HIV ex vivo into mice 9 days after PBMC inoculation to ensure an established infection prior to onset of GVHD. We observed a rapid increase in viral load peaking at a mean of 11 × 10^6^ copies/mL plasma, with no difference between CD4+ inoculation doses (Figure S2C), which led to a rapid decline in the frequency of human CD4+ T cells (Figure S2D). 

**Figure 4:**
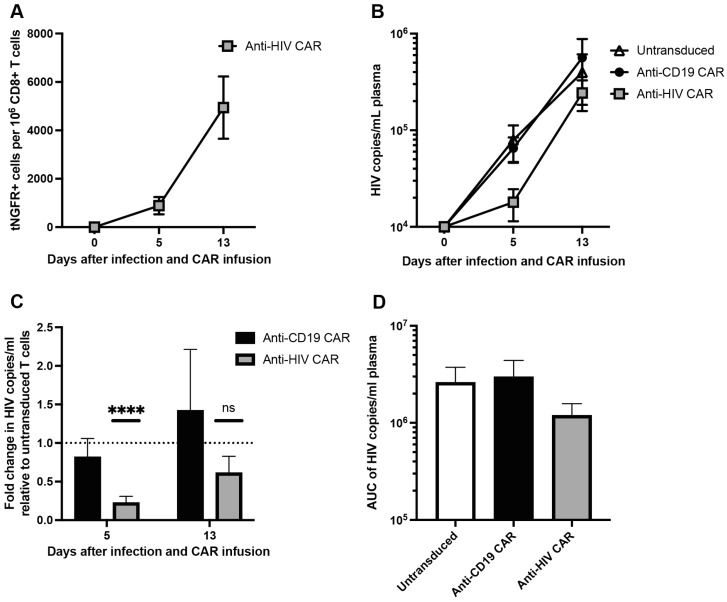
Anti-HIV CAR T cells expand and transiently limit HIV infection in vivo: (**A**) frequency of anti-HIV CAR T cells based on the frequency of tNGFR+ cells per 1 × 10^6^ CD8+ T cells; (**B**) HIV viral load as copies/mL plasma; (**C**) fold change in viral load for each CAR T cell-treated group normalized to the viral load in mice treated with untransduced CD8+ T cells; (**D**) AUC of viral load in (**B**) (n = 7–8 mice from 2 biological donors in each group, data presented as means ± SEM, ns: *p* > 0.05, ****: *p* ≤ 0.0001).

Lastly, we evaluated the expansion and anti-viral effects of the anti-HIV CAR T cells in the humanized mice. We treated the humanized mice with donor-matched CD8+ *AAVS1*-integrated CAR T cells by co-injecting CAR T cells with HIV-infected CD4+ T cells 9 days after PBMC inoculation. Five days after CAR T cell infusion, the anti-HIV CAR T cells were detectable in the peripheral blood (mean 888 tNGFR+ cells per 1 × 10^6^ CD8+ T cells, 95% CI 18–1758) (Figure 4A). However, the frequency of the anti-HIV CAR T cells expanded 5.6-fold (*p* = 0.03) between days 5 and 13 after CAR T cell infusion (mean 4942 tNGFR+ cells per 1 × 10^6^ CD8+ T cells, 95% CI 1633–8250), indicating antigen binding and thus CAR T cell activation (Figure 4A).

Five days after infusion of CAR T cells and HIV-infected CD4+ T cells, mice treated with anti-HIV CAR T cells had a 77.4% reduction in viral load relative to mice treated with untransduced T cells (*p* < 0.0001, 95% CI 57.2–97.6) (Figure 4C). However, 13 days after infusion, the viral load in anti-HIV CAR T cell-treated mice was not significantly different to mice treated with untransduced T cells. The viral load in anti-CD19 CAR T cell-treated mice did not differ significantly from that of mice treated with untransduced T cells at any timepoint (Figure 4C).

Although the anti-HIV CAR T cells caused a lower viral load after five days, the AUC of viral load for each treatment was not significantly different (Figure 4D).

These results demonstrated that the anti-HIV CAR T cells expanded and were thus reactive towards HIV-infected cells in vivo. Furthermore, the anti-HIV CAR T cells showed a transiently limiting effect on HIV infection in vivo but could not retain the effect 13 days after infection.

## 4. Discussion

Because of the immunological similarities between hematological tumors and chronic HIV infection, adoptive immunotherapy using CAR T cells may hold great promise as a curative strategy in HIV infection. However, the approach faces several obstacles, including targeting a rapidly mutating virus and susceptibility to infection of the infused CAR T cells. In the current study, we used a clinically potent bNAb to effectively target HIV-infected cells with CAR T cells. We further engineered the CAR T cells to be resistant to HIV infection by using CRISPR/Cas9-mediated integration of the CAR cassette into the *CCR5* locus.

Several improvements have been made to the safety and efficacy of CRISPR/Cas gene editing and multiple therapies have entered clinical trials [39,40]. When producing CAR T cells, targeted integration of the CAR expression cassette holds several advantages to traditional lentiviral transduction where the transgene is randomly inserted into the genome. Targeted integration with genome editing allows for simultaneous integration of a transgene and disruption of the gene in which the transgene is targeted. This approach has successfully been used to efficiently disrupt the T cell receptor by integrating a CAR into the *TRAC* locus [30,41]. In the current study, the targeted integration of a CAR into *CCR5* rendered the T cells resistant to HIV infection despite 69% INDELs and integration rates up to 20%. We hypothesize that the complete protection from infection was a result of all cells having either bi- and monoallelic disruption of CCR5 since heterozygosity of CCR5 leads to reduced infectability [42]. Importantly, disruption of CCR5 did not alter the cytotoxic capacity of the CAR T cells compared to *AAVS1*-integrated CAR. However, further analyses must be performed on other aspects of T cell functionality, e.g., the memory phenotype. Although we observed complete protection from R5-tropic virus, CCR5 disruption is not expected to confer protection from X4-tropic virus [43].

Antigen escape is a major obstacle to effective CAR T cell therapies [44]. The rapidly evolving nature of HIV may leave anti-HIV CAR T cells vulnerable to mutations that enable escape by the virus. In this study, we used the clinically potent bNAb 10-1074. Despite 10-1074 being highly efficient in targeting HIV, it is susceptible to viral escape during monotherapy since a specific single amino acid mutation will render the virus resistant to the antibody [13,15]. However, a recent clinical trial using a combination of 10-1074 and 3BNC117, a bNAb targeting the CD4-binding site of Env, showed prolonged viral suppression in the absence of ART when serum levels of both bNAbs were sufficiently high [13]. This suggests that combining multiple CAR T cells targeting different conserved epitopes or CAR constructs encoding several targeting domains (bispecific CARs) may be feasible and likely required to mitigate the risk of viral escape mutations and resistance to treatment.

This study investigated CCR5 disruption in CAR T cells to produce an HIV-resistant phenotype of CD4+ CAR T cells that would otherwise become infected and die. Protecting CD4+ CAR T cells from infection will likely increase the potency of the CAR T cell product. However, the models used in this study for assessing cytotoxicity could not evaluate the effect of CD4+ CAR T cells on the cytotoxic capacity of the CAR T cell product. All results of cytotoxicity in vitro and the limiting effects on HIV spreading in vivo were therefore solely due to the effect of CD8+ CAR T cells. However, several studies on anti-CD19 CAR T cells have investigated the effect of defined CD4:CD8 ratios and memory subsets [19,20]. In this study, we observed comparable cytotoxicity between the anti-CD19 CAR T cells and anti-HIV CAR T cells on their respective target cell. Therefore, we expect a similar potency for defined CD4:CD8 ratios and subsets in the anti-HIV CAR T cells as observed in the anti-CD19 CAR T cell studies.

We employed a PBMC humanized mouse model which allowed us to produce CAR T cells donor-matched to the PBMCs used for humanization. The model had a limited experimental time frame due to the onset of GVHD caused by xeno-MHC mismatch between the murine host MHC and donor PBMC HLA-restriction [37]. In humanized mouse models based on hematopoietic stem cells (HSC), T cells are selected and developed in the murine thymus, thereby limiting the development of GVHD [45]. However, the use of HSC-based humanized mice in cellular immunotherapies is limited by the complexity of donor-matching the CAR T cells to HSCs used for humanization. Thus, we opted to use the PBMC humanized mouse model as a practical, time-, and cost-effective alternative to HSC-derived humanized mouse models. Due to the early onset of GVHD in the PBMC humanized mice, we wished to ensure a rapid establishment of HIV infection and therefore infected CD4+ T cells with HIV ex vivo prior to infusion. The ex vivo-stimulated CD4+ T cells were thus actively replicating virus at the time of infusion. Because of the xeno-MHC environment in the mouse, most human CD4+ T cells were highly activated and thereby susceptible to infection from newly released virions [46]. We observed a rapid and robust increase in viral load in the mice at a supraphysiological rate of replication, most likely due to the overly activated state of the human T cell compartment. We hypothesize that the transient nature of the limiting effect on HIV replication observed in the anti-HIV CAR T cell-treated mice could be due to this supraphysiological replication rate, which overwhelmed the anti-HIV CAR T cells 13 days after infection when most human CD4+ T cells had been infected and died. However, we observed a highly significant expansion of the number of anti-HIV CAR T cells 13 days after infection, indicating specific activation of the CAR and thus functionality. We did not observe this expansion in anti-CD19 CAR T cells from 5 to 13 days after infusion, which was likely caused by the anti-CD19 CAR T cells killing the B cells, leading to an absence of antigenic stimulus at day 13 in the anti-CD19 CAR T cell-treated mice. The rate of anti-HIV CAR T cell expansion and subsequent killing of target cells from 5 to 13 days after infection was therefore not sufficient to limit HIV spreading after 13 days in this humanized mouse model. Furthermore, the model does not allow for long term evaluation of HIV infection and the effect on viral reservoirs in ART-treated animals. However, the efficient and specific killing of HIV-infected cells in vitro coupled with in vivo expansion indicated that the anti-HIV CAR T cells potently inhibited HIV infection.

In conclusion, we show that producing anti-HIV CAR T cells by CRISPR/Cas9-mediated integration of the CAR expression cassette for simultaneous CCR5 disruption and CAR integration along with use of an anti-HIV bNAb as the antigen-targeting domain is a feasible approach for developing efficient anti-HIV CAR T cells as a potential curative therapy.

## Data Availability

The datasets presented in this study are available from the corresponding author on reasonable request.

## Supplementary Materials

The following supporting information can be downloaded at: https://www.mdpi.com/article/10.3390/v15010202/s1, Figure S1. INDEL analysis of the CCR5-edit and dose dependent killing of B cells (**a**) INDEL analysis of CCR5-RNP-treated CD4+ T cells; (**b**) dose-dependent cytotoxicity of CAR T cells on autologous B cells after 24 hours of co-culture (data presented as means ± SEM). Figure S2. Optimization of the PBMC humanized mouse model (**a**) Effect on humanization (as ratio of human CD45 to murine CD45) of CD8-depletion of PBMCs prior to inoculation into NOG mice; (**b**) Effect on humanization of cryopreserving PBMCs prior to inoculation into NOG mice; (**c**) Effect on viral load of inoculated HIV-infected CD4+ T cell dose; (**d**) Effect of HIV infection on percentage CD4+ T cells of CD3+ cells (n = 7–8 mice from 2 biological donors in each group, data presented as means ± SEM).
