# Supplementary material for: Development of HIV-Resistant CAR T Cells by CRISPR/Cas-Mediated CAR Integration into the CCR5 Locus
Source: Viruses. 2023 Jan 10;15(1):202. doi: 10.3390/v15010202 (PMC9862650; doi:10.3390/v15010202)
Supplement: Supplementary file 1 [file viruses-15-00202-s001.zip › viruses-2144090-supplementary.pdf]

Supplementary figures

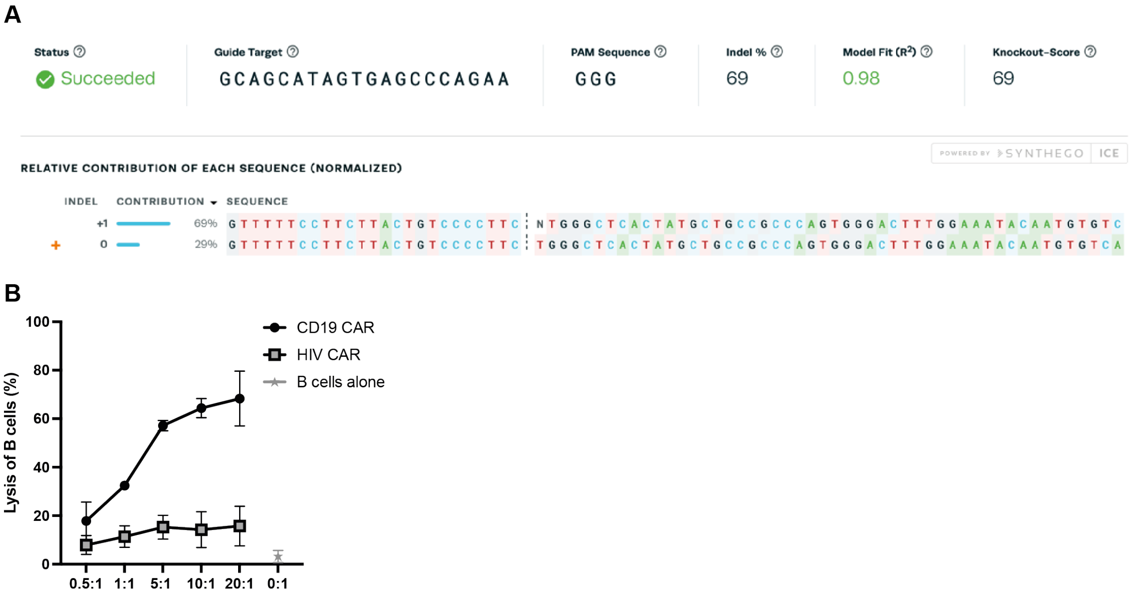

Supplementary Figure S1. INDEL analysis of the CCR5-edit and dose dependent killing of B cells (**a**) INDEL analysis of CCR5-RNP-treated CD4+ T cells; (**b**) dose-dependent cytotoxicity of CAR T cells on autologous B cells after 24 hours of co-culture (data presented as means ± SEM).

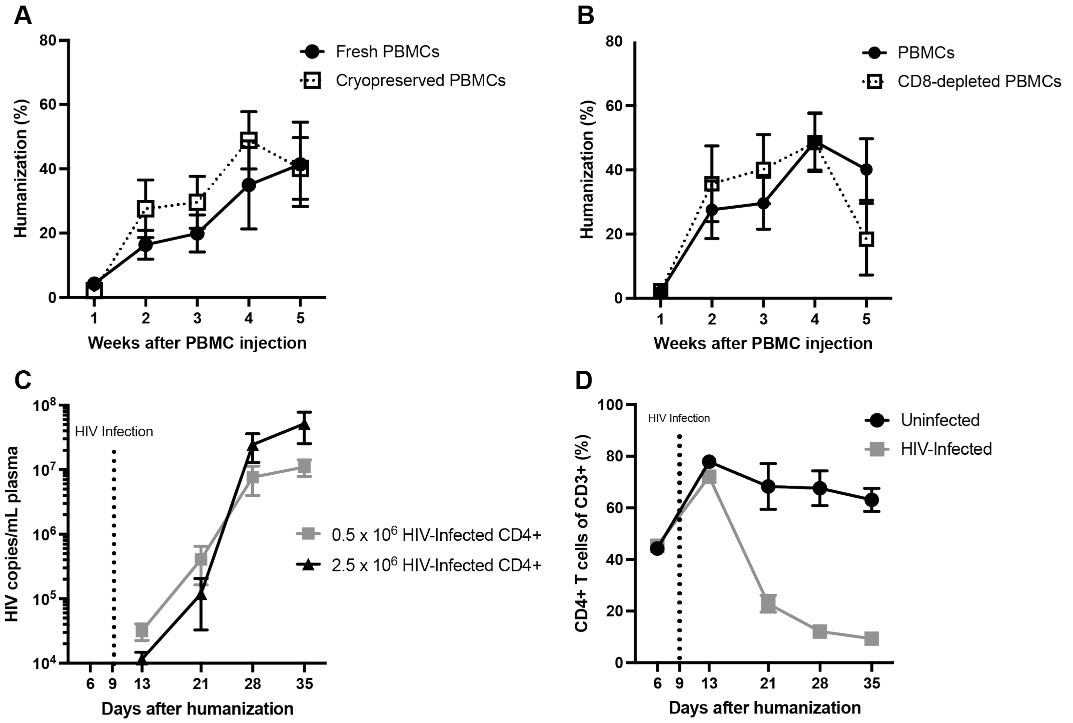

Supplementary Figure S2. Optimization of the PBMC humanized mouse model (**a**) Effect on humanization (as ratio of human CD45 to murine CD45) of CD8-depletion of PBMCs prior to inoculation into NOG mice; (**b**) Effect on humanization of cryopreserving PBMCs prior to inoculation into NOG mice; (**c**) Effect on viral load of inoculated HIV-infected CD4+ T cell dose; (**d**) Effect of HIV infection on percentage CD4+ T cells of CD3+ cells (n = 7-8 mice from 2 biological donors in each group, data presented as means ± SEM).
